# Round the Hospitals

**Published:** 1919-03-08

**Authors:** 


### ROUND THE HOSPITALS.

At the Investiture held at Buckingham Palace on March 1 the King conferred the decoration of the Royal Red Cross, First Class, on Sister Jeane Taggart, Q.A.I.M.N.S. (R.). His Majesty conferred the Second Class of the Order on Sister Margaret Heggie, Q.A.I.M.N.S. (R.); on Matron Mary Smith, Sister Helen Morton, and Sister Agnes Quinn, of the Civil Nursing Service; on Sister Maggie Jackson, B.R.C.S.; on Sister Jessie Baxter, Sister Emma Cuthbert, Sister Minnie Mears, Sister Christense Sorenson, Sister Ethel Soutar, Staff-nurse Gladys Jarrett, Staff-nurse Ethel Merde, and Staff-nurse Lilian Rutherford, all of the Australian A.N.S.; also on Mrs. Marguerite Clement, Mrs. Mary Hewlett Johnson, and Miss Rita Pitts, V.A.D. Miss Winifred Elwes, V.A.D. ambulance driver, had the honour of receiving the Military Medal from the King.

At the Investiture held on February 26 in the Ball-room of Buckingham Palace the King personally conferred the Royal Red Cross (First Class) on Sister Louisa Stobo, Australian Army Nursing Service, and the Second Class of the Order on Sister Ethel Butler and Sister Elizabeth Geoghegan, both of the Australian A.N.S. All three ladies

**ROUND THE HOSPITALS—(*continued*).**

were subsequently received by Queen Alexandra at Marlborough House, where Dame Ethel Becher was also present. Miss Emma Byles, late matron of Camberwell Infirmary, has been personally decorated by the King with the insignia of a Member of the Order of the British Empire. Florence, Lady Belfield, of the East African Force Nursing Service, has been appointed a Commander of the Civil Division of the Order of the British Empire, in lieu of her previous appointment as an officer of the Order, which is cancelled.

---

Mrs. E. M. Field has been appointed the Chairman of the Committee set up by the College to consider and report upon the conditions of employment of nurses and the salaries paid to them. Mrs. Field has had a wide experience in matters relating to subjects with which this Committee must deal, and our congratulations go out to the Council that the responsible position of Chairman has been placed in such competent hands.

---

Brains are at work in the Infirmaries Committee of the Birmingham Board of Guardians. The Board have been blessed with a very capable matron in their convalescent home. Taking the weekly cost per head of food for the three months October, November, and December in 1915 and in 1918, they find that it worked out at 6s. 2d. and 7s. 6d. respectively, thus showing an increase of 21.6 per cent. only—a truly wonderful achievement. That the convalescents have not suffered is proved by the weighing system in force in the establishment, the inmates on an average having put on weight during the later period at the rate of nearly $\frac{1}{2}$ lb. a week. The following figures show the comparative cost and consumption of the main articles of diet:—

| | | Per head per week. | Cost. |
|---|---|---|---|
| 1915 | Meat | $1\frac{2}{5}$ lb. | 1/3 |
| 1918 | ,, | 1 lb. | 1/11 |
| 1915 | Milk | 4 pints | $7\frac{1}{3}d.$ |
| 1918 | ,, | $3\frac{1}{2}$ ,, | 1/1 |
| 1915 | Bread | $6\frac{1}{2}$ lb. | 1/- |
| 1918 | ,, | $3\frac{2}{3}$ lb. | $8\frac{1}{2}d.$ |

The regulations of the Food Controller had to be observed, but the meals were kept at a level of good nutritious quality, and were constantly varied. The Committee have put on record their appreciation of the matron's excellent qualities as housekeeper and the services she has rendered in the management of the home. We should like to know her name.

---

We have recently had brought to our notice by an old correspondent the admirable nursing in the Royal Edinburgh Hospital for Incurables. This aspect of the work done at Longmore Hospital was brought before the subscribers very forcibly at the annual meeting by Dr. Blaikie, who spoke of the devotion which nursing of this type demanded from the staff. "Without the incentive of hoping for final recovery, they must go on from day to day and from year to year cheering, guiding, comforting; often, I have no doubt, meeting with much impatience and irritation, yet ever helpful, ever comforting." Dr. Blaikie went on to say that in his eighteen years of hospital experience nothing has struck him more than the overwhelming importance of the nurse's work, and nothing had forced his admiration more than the way in which that work was done. There was no hope of fame, and no demand for it. "Carry on" had been the motto of the whole nation during the war, but it was the motto of the nurse through her whole life, and this on a pittance which would be scorned by, say, a munition worker, and with hours that would be repudiated by an artisan.

---

The Scottish branch of the British Red Cross Society are not in entire sympathy with the Devonshire House V.A.D. scholarship scheme, and propose to form an independent scheme under a Scottish Red Cross Nursing Fund. Sir George Thomas Beatson, M.D., K.C.B., K.B.E., D.L., has outlined their proposed policy in an interesting little pamphlet, from which it appears that the Scottish branch strongly dissents from the notion that only V.A.D. workers should participate in the scholarship scheme. He holds that trained nurses ought to be included in the scope of the scheme; that women who are debarred from taking hospital training on account of the necessity for contributing to the upkeep of their own homes ought to be given grants in aid; that a Red Cross Nursing Reserve should be gradually built up; that the civil nursing training-schools ought to be helped by grants given towards the expense of a sister-tutor and by the founding of Red Cross nursing prizes; lastly, that Red Cross lecturers and examiners should be appointed—Navy, Army, and civilian sisters and matrons being eligible for such posts. He reckons that a capital sum of £50,000 would be required to carry out these proposals.

---

Edinburgh has been in the grip of a deadly recurrence of influenza. Miss Graham, of the Scottish Association of Trained Nurses, had twenty applications for nurses in one day, and could only supply three. Seven other nursing institutions in Edinburgh are in a similar plight. The Medical Officer of Health has applied for nurses to be demobilised to meet this extremity, but so far without result. They are offering from £1 to 25s. a week to V.A.D. workers who will volunteer to help in this extremity. Several Edinburgh hospitals are working under-staffed owing to their nurses being laid low, and the epidemic appears to be fully as virulent as that in the autumn.

---

Taunton Red Cross Hospital is one of those establishments where no friction occurred between members of the trained and voluntary staffs, but goodwill prevailed all round. It has now wound up its affairs with a farewell gathering of staff, helpers, and V.A.D. men. A diamond brooch was given to the Vice-President, Miss Janet Vaughan, O.B.E., on whom fell the labour of organisation,

**ROUND THE HOSPITALS—**

and to the commandant, Mrs. Gerald Fowler. The matron, Miss Fry, received a silver inkstand from the sisters, nurses, and other members of the staff in token of their regard and appreciation of the happy relations that had existed between them, and she also received a silver frame from the men's V.A.D. as a token of their esteem and gratitude.

The North Riding Nursing Association has been unfortunate during the past year in the matter of illness among the nurses, owing mainly to extra pressure of work and a depleted staff. The total on the books is now fifty-one, including district nurses and probationers, and probably work could be found for half as many again. Miss Goodrich, who has been lady superintendent for seven years, has now been appointed matron of the Stamford, Rutland, and General Infirmary. She has done admirable work for the North Riding Association, the work of which has grown and developed considerably under her able management. Latterly, owing to the appointment of her assistant superintendent, Miss Briscoe, to the post of assistant matron at Addenbrooke's Hospital, Cambridge, she has been working single-handed, and has narrowly escaped a breakdown. These war years will be remembered ruefully by the managers of all nursing associations, and the nurses who have held the fort ought not to be forgotten when honours are dealt out.

The West Kent General Hospital, Maidstone, is revising salaries on a liberal scale. The sisters have hitherto been receiving £40, £41, according to the capacity in which they serve. Three of the sisters are now to receive £45, rising by yearly increment of £2 10s. to £55; three will commence at £50 and rise to £60. Probationers are to receive £20, £25, and £30, in their first, second, and third years respectively, in lieu of £11, £15, and £18. Fourth year nurses are to receive £40. District nurses are attached to the staff, and we welcome the announcement that those who live out are for the future to receive £105 inclusive, in lieu of £80. District nurses who live in continue to receive £40 a year, but rise by yearly increment of £2 10s. to £45. The most important change, however, is the increase of the matron's salary from £100 to £200. By this very wise policy the Board of Management stand to gain a great accession of strength. Readers will note in our advertisement columns that the post of matron is now vacant, and we feel assured that the announcement will attract applicants of wide attainments in directing the training of nurses, and in the control of such institution matters as fall within the matron's scope. The post is an unusually interesting one, and with the support of a board bent on a forward policy much may be accomplished.

It will be thought incredible that the War Office should be engaging unqualified masseuses and masseurs, but the Almeric Paget Massage Club have brought into the open and formally protested against a recent Army Council Instruction authorising these persons to enter the service, and moreover penalising immobile qualified masseuses by directing that they shall lose their pay on becoming sick. The masseuses demand to be considered "professional women," entitled to special medical benefits and travelling facilities; that the yearly increment in pay be increased to 5s., and that full sick pay be granted for six weeks to those in the immobile category. The War Office has never dealt liberally with the masseuse from the beginning of the war, and this in spite of overwhelming testimony to the curative effects of massage on the wounded and nerve-stricken soldier. It is high time this arm of the health service was strengthened by offering the just conditions which can alone attract women of high calibre into the service. Nothing short of exact training, combined with true intelligence, will do in this work. Unqualified women are worse than useless, they are actually harmful, and if the Army authorities do not yet understand this they should consult the people who do.

We observe the mention of various bodies which have obtained representation on the Women's Committee in connection with the Ministry of Health, but so far we have no evidence to show that the College of Nursing is one of them. It is highly important that representatives of the College should be available to afford accurate information on nursing organisations when required, and no time should be lost before entering into relations with those responsible for the constitution of this important committee. Even the doctors themselves will hardly have more responsibility thrown on them by the new Health Bill than nurses.

Harrow has shown great public spirit in already completing and equipping its War Memorial Maternity Hostel at a cost of £3,000. Lady Rhondda performed the opening ceremony on March 1, and she expressed her opinion that nothing of more value was being done in the country at the present time than that in connection with maternity hostels. The hostel is not intended for Poor-Law cases, but is intended as a kind of half-way house between lying-in hospitals in large towns and the expensive nursing homes.

A somewhat intricate arrangement as to working hours and leave has been arrived at by consent in the works of Messrs. Cadbury Brothers, Limited, at Bournville, which might prove suggestive in the matter of nurses' hours. The standard rate of hours is worked out for a month of four weeks, and averages forty-four hours a week, with one full Saturday in every four. On Sunday, of course, no work is done. The decision was arrived at through the action of two work councils composed of men and women respectively. Three proposals were formulated and laid before all the workers. A referendum was then conducted on the alternative vote principle, and the majority were in favour of the above arrangement. The principle of one day in seven ought, it is generally conceded, to be

**ROUND THE HOSPITALS—(*continued*).**

combined with the provision of an extra day once a month, in order to enable nurses to get away from hospital for a couple of nights.

The newly founded Priory for Wales of the Order of the Hospital of St. John of Jerusalem was inaugurated at Cardiff on March 1, under the presidency of Sir Owen Philipps, M.P. Recalling the foundation of the Order when Christianity was yet young, for ministering to the sick and wounded, the President said it was their aim to carry on a living organisation for the benefit of the Welsh people, based on self-governing and democratic principles. There is to be close co-operation between the Chapter of the Priory and other bodies engaged in levelling up the health of the community, particularly, we are glad to learn, with the nursing associations throughout the Principality. We have continual evidence of the fine organisation of the County Nursing Associations in Wales, and trust their position will be strengthened by the support of this newly formed body.

At the annual meeting of the Stroud Nursing Association good progress was reported all along the line. The Association employs not only visiting district nurses, but cottage nurses, who stay in the homes of patients unable to pay full fees. They have also a home in which patients can be received. Dr. Seymour, speaking from personal experience of the nurses' work during the last three years, bore warm testimony to their "kindness, energy, sympathy, and zeal—virtues which were identical with the fruits of the Spirit, and revealed the Divine influence in their work and conduct." He thanked the lady superintendent, Miss Berks, for her willingness to undertake duties which had often lightened his own responsibilities.

Nurse Nichols, who has worked under the Walsall Victoria Nursing Institution for ten years, was presented by the Mayor of Walsall with a medal at the recent annual meeting of the institution. The superintendent, Miss Holloway, reported that at the height of the influenza epidemic in November the six nurses created a record by paying 4,308 visits to the homes of patients—over 1,000 a week.

We have received an interesting letter (too long for publication) from a trained nurse with very varied experience, "One Who is Loyal," making some practical suggestions. She urges, first, that the foster-mothers in charge of Poor-Law children's homes should be trained nurses, of higher status than the people at present available. We gather that she thinks it might be possible to bring the children's homes under the influence of the nurse-training school. She urges that no person be placed in authority who is not properly qualified, because "it is impossible to be just in detail without going through the mill." She is on strong ground in urging that the aged and senile should all be under trained care and supervision, and that the numbers dealt with by each nurse should be lessened. We feel sure she is correct in stating that "impossible numbers" are responsible for making nurses callous. We hope much from the transfer of the aged from the grip of the Poor-Law, which has ever dealt hardly and unintelligently with them. It is a great pleasure to find nurses thinking out these matters and reaching sound conclusions.

The Deptford Health Centre has issued its ninth report in the form of a little pamphlet, price 6d., to be obtained of P. S. King and Son, Orchard House, Westminster. Its contents comprise particulars relating to the Dental Clinic, the Baby Camp and Training Centre, the Minor Ailment Department, and the Bathing Centre, where 8,000 children were treated in the year. The Baby Camp averages an attendance of eighty-five, from infants to children of seven, with seventeen nurse teachers in training from schools in different parts of the country. There are also twelve students, ranging in age from sixteen to twenty-five years, mostly taking a two-year course. Everything is done in the open air, winter and summer, and at no moment in the year is the health so satisfactory as in mid-winter. Over 75 per cent. of the babies are rickety on entrance; 50 per cent. are verminous. All make good recoveries, and "the new-comers are the only pale and unhappy children." The course of instruction is valuable for infant school teachers, V.A.D. and welfare workers, and the pamphlet should be widely circulated.

The standard of education for Natal probationers is to be raised. Dr. Dumat, at the meeting of the Natal Medical Council last December, brought to light as an examiner the frequent instances of poor education which came under his notice, the "dreadful spelling and exceedingly faulty style of expression and diction," often displayed by women bearing distinctly British names. He urged the adoption of the sixth standard as the minimum of primary education to be demanded from probationers, but with the proviso that a fifth standard in arithmetic should suffice. It does not seem a very drastic proposal, and we trust it may meet with acceptance. The important thing is to get the principle of an educational standard accepted. After that is done it can be raised whenever the schools begin to turn out better pupils.

During the year 1917-18 the New Zealand Board held two examinations under the Registration Act, and passed 161 candidates out of 193. Besides this, fifteen nurses were registered from overseas. The number of nurses enlisted on military service reached the total of 511. During the year orders were received from the War Office that, owing to the submarine danger, sisters were not to proceed on hospital ships; male orderlies were to nurse the sick and wounded on board instead. This was found so unsatisfactory, not only in New Zealand ships, but in all ships, that the sisters were allowed again to share the risks, from which they themselves had never shrunk.

